# A web-oriented software for the optimization of pooled experiments in NGS for detection of rare mutations

**DOI:** 10.1186/s13104-016-1889-6

**Published:** 2016-02-17

**Authors:** Daniela Evangelista, Antonio Zuccaro, Algirdas Lančinskas, Julius Žilinskas, Mario R. Guarracino

**Affiliations:** LabGTP (Laboratory of Genomics, Transcriptomics and Proteomics), Institute for High Performance Computing and Networking (ICAR), National Research Council (CNR), Via Pietro Castellino 111, 80131 Naples, Campania Italy; Department of Computer Science, University of Naples Parthenope, Via Amm. F. Acton, 80133 Naples, Italy; Institute of Mathematics and Informatics, Vilnius University, Akademijos 4, 08663 Vilnius, Lithuania

**Keywords:** Pooled experiments, Rare mutation, Next generation sequencing, High-throughput data

## Abstract

**Background:**

The cost per patient of next generation sequencing for detection of rare mutations may be significantly reduced using pooled experiments. Recently, some techniques have been proposed for the planning of pooled experiments and for the optimal allocation of patients into pools. However, the lack of a user friendly resource for planning the design of pooled experiments forces the scientists to do frequent, complex and long computations.

**Results:**

OPENDoRM is a powerful collection of novel mathematical algorithms usable via an intuitive graphical user interface. It enables researchers to speed up the planning of their routine experiments, as well as, to support scientists without specific bioinformatics expertises. Users can automatically carry out analysis in terms of costs associated with the optimal allocation of patients in pools. They are also able to choose between three distinct pooling mathematical methods, each of which also suggests the optimal configuration for the submitted experiment. Importantly, in order to keep track of the performed experiments, users can save and export the results of their experiments in standard tabular and charts contents.

**Conclusion:**

OPENDoRM is a freely available web-oriented application for the planning of pooled NGS experiments, available at: http://www-labgtp.na.icar.cnr.it/OPENDoRM. Its easy and intuitive graphical user interface enables researchers to plan theirs experiments using novel algorithms, and to interactively visualize the results.

**Electronic supplementary material:**

The online version of this article (doi:10.1186/s13104-016-1889-6) contains supplementary material, which is available to authorized users.

## Background

Next generation sequencing (NGS) is a recent approach that has begun a real revolution in genomics. It allows researchers to study biological systems to a level hitherto impossible, enabling numerous groundbreaking discoveries such as detection of rare causative mutations involved in genetic diseases [1]. Nevertheless, independently from the platforms used, its widespread diffusion is inhibited by the remarkable cost [2]. A possible solution is to pool more samples together, although subsequent Sanger sequencing is needed for the assignment of the mutation to the patient. The detection of rare mutations in individual patients grouped into pools could be more efficiently discovered. Indeed, the pooling techniques are aimed to examine a set of DNA samples from a group of individuals in order to ascribe the identified mutations to a specific patient. A classical protocol dedicated to the detection of mutation defines that for each individual patient to be tested, each exon—or few closely located exons—is PCR amplified and then assayed [3]. In light of these considerations, in the statistical literature, is easy to find a large number of papers which refer to the usage of pools or groups of samples to identify individuals or to estimate the prevalence of such a rare characteristic [4–7] in literature, the lack of user-friendly software makes difficult for researchers to plan pooled NGS experiments without consulting a large number of of papers [8–10] to find the best method for their own needs, or without performing troublesome computations to evaluate the costs. We propose OPENDoRM (optimization of pooled experiments in NGS for detection of rare mutations), a new web tool for planning NGS experiments with a simple graphical user interface (GUI). It provides flexibility to the users for automatically carrying out analysis in terms of costs associated with the optimal allocation of patients in pools, suggesting also the optimal configurations of their experiments. The OPENDoRM structure can be split into four components: (i) global settings for the NGS experiment; (ii) data processing; (iii) visual exploration; (iv) data interpretation. It is able to: (i) describe the pooling of high-throughput generated data using four different algorithms; (ii) identify the optimal number of patients in each pool with respect to minimization of the cost of the experiment; (iii) generate easy-to-read reports and charts for better understanding the planning of the experiments.

## Methods

### OPENDoRM design

Leading studies using pooled experiments in several genetic and genomic applications can be found in [8–10] . Nevertheless, their limit is that no evaluation has been done to assess the group size of the pools and the associated cost with the experiment and its biological validation. OPENDoRM is the first all-in-one web resource for planning pooled NGS experiments with or without control pools. Its structure consists of eight main sections. The *Pooling section* represents the web portal core. From this section, users can access to the *Methods* list page in which three distinct strategies are implemented: (i) without Replica; (ii) with Replica and (iii) Hybrid; and four algorithms are present: (i) NoReplica; (ii) OptReplica; (iii) Transposition and (iv) DiagWalks.

### Algorithms description

Although the original algorithms of the first two strategies are inspired and thoroughly described in a previous work [11] we believe is useful to provide the main details of each of them, before to introduce the Hybrid algorithm, named DiagWalks, which has been specifically developed for this web-oriented software.

Since we propose a technique to plan NGS pooled experiments, we consider worthwhile take into account experimental setting-out without or with replication of the patients. NoReplica belongs to the Without Replica strategy. Here, each patient might bring at most $N_{m}$ rare mutations. All n patients can be allocated in p pools consisting of $m_{1}$, … , $m_{p}$ patients and where each pool is restricted by a maximum number mmax of patient. In this case all found mutations need to be assigned to patients present in the pool. OptReplica and transposition belong to the With Replica strategy. The first one can be described as: allocate each patient in both the first pool that is not yet completely filled, and in the first pool with the smallest number of allocated patients. The second one is based on the concept of transposition matrix where patients are properly distributed into main pools and replicated pools. This approach can be applied if the maximum number of patients allowed in a single pool is greater than or equal to the number of main pools and by taking into account the two constraints: (i) the number of replicated pools cannot be larger than the total number of patients; and (ii) the number of replicated pools cannot be smaller than number of patients in the largest main pool.

### DiagWalks algorithm

DiagWalks is a hybrid method since, respect to the previous algorithms, it exploits both control pools (i.e. OptReplica and Transposition methods) and Sanger tests (i.e. NoReplica method). The main goal of DiagWalks is to start the sequencing of pools as soon as patient’s samples are available while not increasing significantly the overall costs (p$_{m}$+p$_{c}$) $\cdot$ c$_{1}$+(pat $\cdot$ c$_{2}$). The total cost $C_{T}$ of the experiment is calculated as:

1 $$\begin{aligned} C_{T} = (p_{m} + p_{c}) \cdot c_{1} + \sum _{j=1}^{n_{mut}}c_{2} \cdot N_{m} \cdot pat^{2}_{j} \end{aligned}$$

where $j \,=\, 1,2, \ldots ,n_{{mut}}$ is the number of control pools in which there are more than one patient in common with any of the main pools, $p_{m}$ is the number of main pools, $p_{c}$ is the number of control pools, $c_{1}$ is the cost of a single pool for NGS, $c_{2}$ is the cost of a single Sanger test, $N_{m}$ is the number of mutations to detect and $pat_{j}^2$ is the number of patients in common between a main pool and j-th control pool elevated to the second power.

The workings of DiagWalks is moving diagonally upwards, from left to right, along the main pools matrix, each time replicating the current patient inside the control pools matrix, which is scanned moving from the top to the bottom along the rows and moving from left to right along the columns. It can be summarized as follows:

1. The starting point of DiagWalks is always the top left corner (1,1) of the main pools matrix. This patient is replicated in position (1,1) of the control pools matrix
2. The scanning sequence moves onto position (2,1) of the main pools matrix. This patient also gets replicated in position (2,1) of the control pools matrix
3. At this point, the first diagonal walk begins. The scanning sequence continues moving diagonally upwards moving from left to right. Position (1,2) of the main pools matrix is reached. This patient is inserted in position (2,1) of the control pools matrix

Each diagonal walk stops when one of the two following conditions is met:

1. The number of patients already inserted in a control pool is equal to the poolsize (i.e. three patients already inserted in a control pool with its poolsize being equal to three). In this case, the scanning sequence will restart from the first patient who has not been replicated yet who can be found scanning the main pools matrix starting from position (1,1) and moving from the top to the bottom along the rows and from left to right along the columns. Once this patient is found and replicated, the scanning sequence will move diagonally upwards along the main pools matrix, the first patient of each new scanning sequence being inserted into a new control pool
2. The number of patients already inserted in a control pool is less than the poolsize (i.e. three patients already inserted in a control pool with its poolsize being equal to five). The current position of the scanning sequence in the main pools matrix, however, is such that it does not allow for a diagonal walk either because it would get outside the “bounds” of the matrix or because it would end up on a patient who has already been replicated. In this case, the scanning sequence restarts from the first patient who has not been replicated yet who can be found scanning the main pools matrix moving from the current position from left to right and from the top to the bottom.

Finally, the following strategy is adopted: if the number of patients who still need to be replicated is greater than the remaining empty locations of the control pools matrix, a new control pool is added. If, instead, the number of patients who still need to be replicated is less than the remaining empty locations of the control pools matrix, they get replicated along the same control pool.

**Fig. 1:**
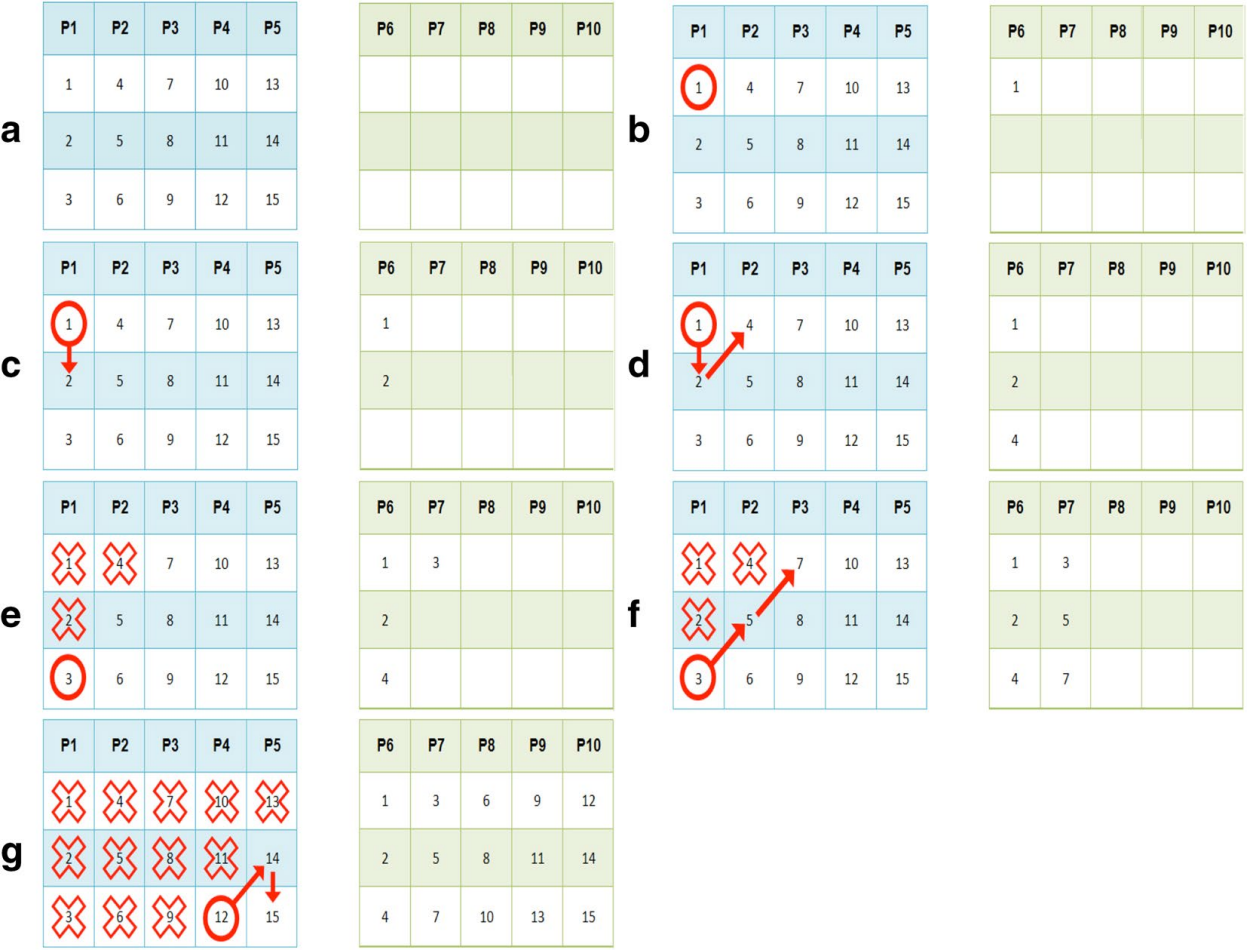
DiagWalks algorithm steps. A specific example to show the behaviour of the algorithm on a court of 15 patients and a poolsize equal to 3

Let us assume we have a court of 15 patients and a poolsize equal to 3 (see Fig. 1a). The starting condition is the following: (i) the first step is starting from patient 1 in the main pools matrix and replicating it in the exact same position in the control pools matrix (see Fig. 1b); (ii) the next step is moving onto patient 2 of the main pools matrix and replicating it in the exact same position in the control pools matrix (see Fig. 1c); (iii) the first diagonal walk begins, scanning the main pools matrix moving diagonally upwards from left to right. The sequence ends up on patient 4 of the main pools matrix, which is replicated in the final empty location of P6 (see Fig. 1d); (iv) condition 1 explained before is met: the control pools has no more empty locations. The scanning sequence will restart from the first patient who has not been replicated yet and who can be found scanning the main pools matrix starting from position (1,1) and moving from the top to the bottom along the rows and from left to right along the columns. Patient 3 is found and replicated in the first empty location of P7 (see Fig. 1e); (v) the main pools matrix is scanned moving diagonally upwards, thus finding and replicating patients 5 and 7 (see Fig. 1f); (vi) by replicating patients in this way, the control pools matrix is therefore completed (see Fig. 1g).

**Table 1:** Comparison of the performance of the four algorithms implemented in OPENDoRM

	NoReplica	OptReplica	Transposition	DiagWalks
Num Patients	5	5	6	6
Num pools	13	22	22	23
Num Sanger test	1580	0	0	20
Expected patient	64	64	56	25
Total cost (€)	2640	22,000	22,000	23,160
Num patients	4	5	6	6
Num pools	32	22	22	23
Num Sanger test	3584	0	0	20
Expected patient	128	128	113	49
Num patients	3	20	20	20
Num pools	667	200	200	200
Num Sanger test	119,960	0	0	160
Expected patient	2000	2000	1901	361
Total cost (€)	1,626,680	200,000	200,000	201,280

Case study 1: The input parameters are: number of patients = 64; max Poolsize = 6; number of mutations to detect = 5; cost of a single Sanger test = €8; cost of a single pool for NGS = €1000. Case study 2: The input parameters are: number of patients = 128; max Poolsize = 8; number of mutations to detect = 7; cost of a single Sanger test = €10; cost of a single pool for NGS = €1000. Case study 3: The input parameters are: number of patients = 2000; max Poolsize = 20; number of mutations to detect = 20; cost of a single Sanger test = €8; cost of a single pool for NGS = €1000

In order to appreciate the benefits of DiagWalks — as evidenced by experiments carried out during the testing phase (Table 1) — a suitable example is represented by the third case study in which a court of 2000 patients, the capacity of a single pool equal to 20 and 20 expected mutations were used.

### The web-oriented software description

**Fig. 2:**
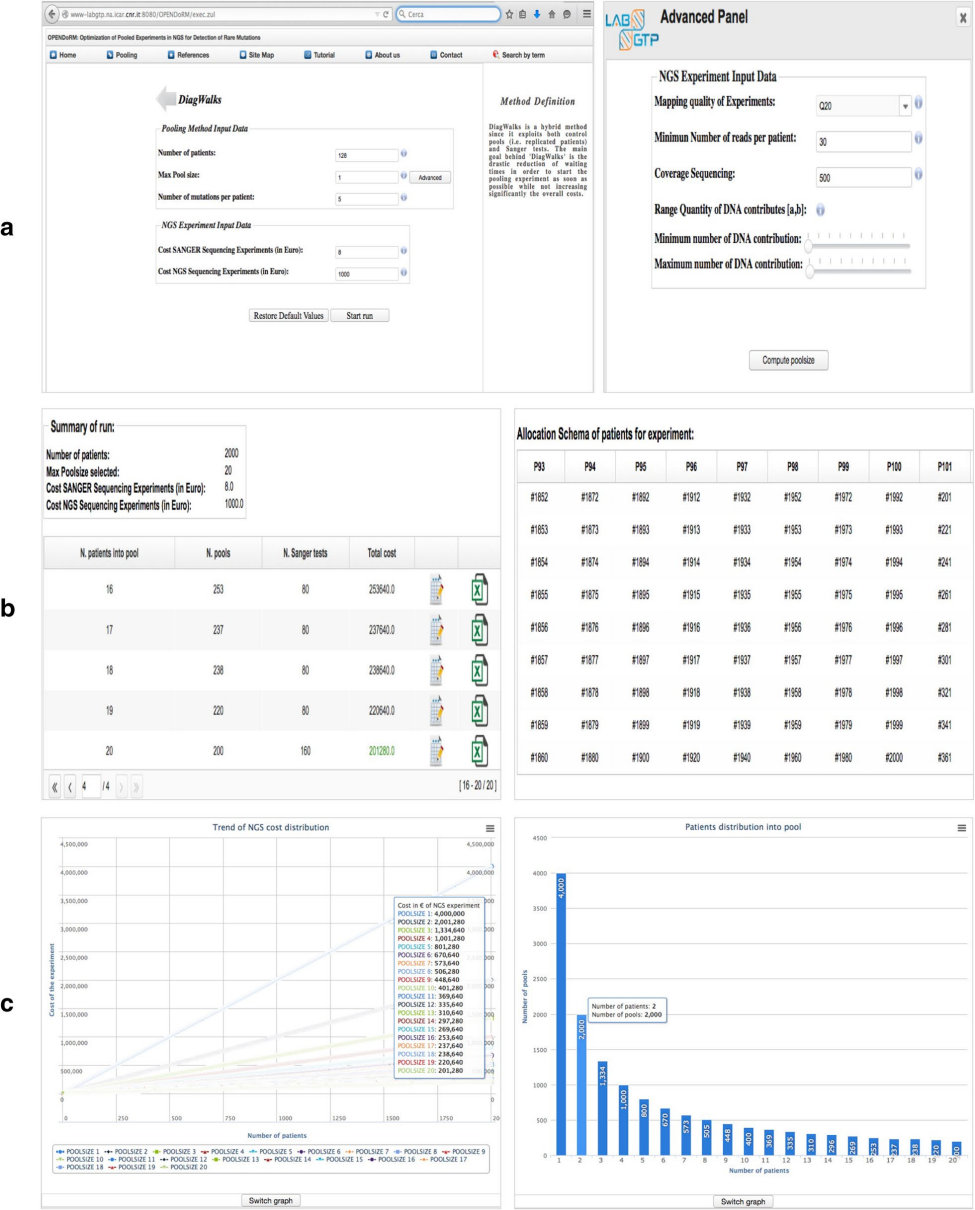
Summary of the final setup produced by DiagWalks algorithm within OPENDoRM interface. **a** DiagWalks algorithm input form with advanced panel in evidence; **b** the results page; **c** plots’ examples: the NGS cost distribution and the patients allocation into pools.

For the setting of the experiment we offer several options, which can be easily modified via textboxes and buttons. The input data depend on the selected methods, the proposed Parameter Box is split in two parts (see Fig. 2a), the first one containing: (i) number of patients; (ii) max pool size; (iii) expected number of mutation per patient; (iv) sanger cost and (v) NGS cost. An advanced panel for skilled users has also been developed. In order to refine the outcomes of the experiment, it is possible to customize the parameters settings by considering the second parameters box: (vi) mapping quality of experiments; (vii) minimum number of reads per patient; (viii) coverage sequencing; (ix) range quantity of DNA contributes. The user can choose to modify certain or all of the above-mentioned parameters and, depending on the selection, a different result is returned. In order to guide the user and provide plausible outcomes, all the parameters are enclosed into tolerance intervals, with the exception of (ii–iii) parameters which can be manually typed on the basis of specific needs. Once the simulation is over, the user is provided with a complete overview of the results viewable in the summary of run and allocation schema of patients windows (see Fig. 2b). In the first one, depending on the input parameters, the system returns all the possible configurations of patients’ distribution into pools, with the related costs. The best configuration is automatically highlighted in green. The other panel shows the way in which the system has arranged the patients for that specific configuration, which can be consulted in detail by clicking the related button. OPENDoRM provides easy-to-read tables and interactive charts for better understanding the results (see Fig. 2c). Users can export the results of their experiments in xls format for tabular contents and png/jpg/svg/pdf for the charts. Moreover, we provide an in-depth user manual of operating principles of the methods (see Additional file 1).

### The implementation

The software has been implemented in a modular way, therefore, it can also be adopted by scientists with low expertise in the design of pooled NGS experiments. The OPENDoRM application has been developed using the ZK framework [12] and J2EE [13] (Java 2 Platform Enterprise Edition) technologies. ZK framework and Ajax technique with XUL/XHTML (XML user interface language/eXtensible hypertext markup language) have been used to design the GUI, taking advantage of their widely used toolkits [14]. The charts displayed at the end of each pooling method simulation were created using ZK charts, which makes visualization of data easy to understand for the end users. It is fully integrated with ZK, thus allowing for a complete control over charts in pure Java.

## Results

### Validation and testing

The current version of OPENDoRM implements three algorithms and features a new one, DiagWalks. It significantly reduces the waiting times—that is, the starting of NGS sequencing between one group of patients and the next one—without creating a considerable economic gap with the other considered methods [11]. The results obtained for, respectively, NoReplica, OptReplica, Transposition and DiagWalks are reported in Table 1. It can easily gather that if, on one hand, the DiagWalks algorithm easily evaluates large courts of patients, on the other hand, it also provides a significant improvement in terms of reducing waiting times. Indeed, it is only necessary to wait for 361 patients’ samples against the 1901 proposed by the Transposition algorithm, while for OptReplica it is always necessary to wait for all samples. The considerably high costs required by NoReplica make the choice of this methodology the least preferred. The additional charge of 1280 € needed by DiagWalks planning (which, as has been evidenced by other case studies, can be quite lower depending upon the input data) is considered negligible compared to the benefits obtained in terms of reduction of waiting times.

The results achieved through the usage of this powerful software can be a springboard for helping scientists in addressing the problem of detecting rare causative mutations in pooled experiments [15].

## Conclusion

OPENDoRM is the first web tool for planning of pooled NGS experiments. Written in a modularized style, it can be easily expanded and can provide flexibility to the users for automatically carrying out analysis in terms of costs associated with the optimal allocation of patients in pools. Users are able to choose between three distinct mathematical methods—*Without Replication, With Replication* and *Hybrid*—each of which also suggests the optimal configuration of the sequencing experiment. The results cannot be compared neither with others obtained in the past nor with other scientific articles since, to the best of our knowledge, in literature there is no other tool with the same aim. For these reasons, OPENDoRM represents a completely innovative approach.

The web resource will be regularly updated on the basis of the progress of our study.

## Availability and requirements

- Project name: A web-oriented software for the optimization of pooled experiments in NGS for detection of rare mutations
- Project home page: http://www-labgtp.na.icar.cnr.it/OPENDoRM
- Operating system(s): Platform independent
- Programming language: Java and XHTML
- Other requirements: no requirement needed
- License: no licence needed
- Any restrictions to use by non-academics: no restriction needed

## Availability of data and materials

All supporting data are included within the manuscript and its additional files.

## Additional file

10.1186/s13104-016-1889-6OPENDoRM user manual. OPENDoRM user’s manual provides detailed case studies with simulated data and illustrates how to use the OPENDoRM algorithms.

## Abbreviations

OPENDoRM: optimization pooled experiments NGS for detection of rare mutations
DNA: deoxyribonucleic acid
PCR: polymerase chain reaction
NGS: next generation sequencing
GUI: graphical user interface
J2EE: java 2 platform enterprise edition
XUL: XML user language
XHTML: eXtensible hypertext markup language
