# Supplementary material for: A web-oriented software for the optimization of pooled experiments in NGS for detection of rare mutations
Source: BMC Res Notes. 2016 Feb 17;9:111. doi: 10.1186/s13104-016-1889-6 (PMC4756526; doi:10.1186/s13104-016-1889-6)

OPENDoRM
USER MANUAL

Updated on January 2016

How to navigate OPENDoRM

The following pages are a schematic representation of how to navigate through OPENDoRM web server in order to extract information of interest. OPENDoRM core is represented by the *Pooling* section. Starting from the *Home page* the user clicks on the related button on the top of the menu bar.

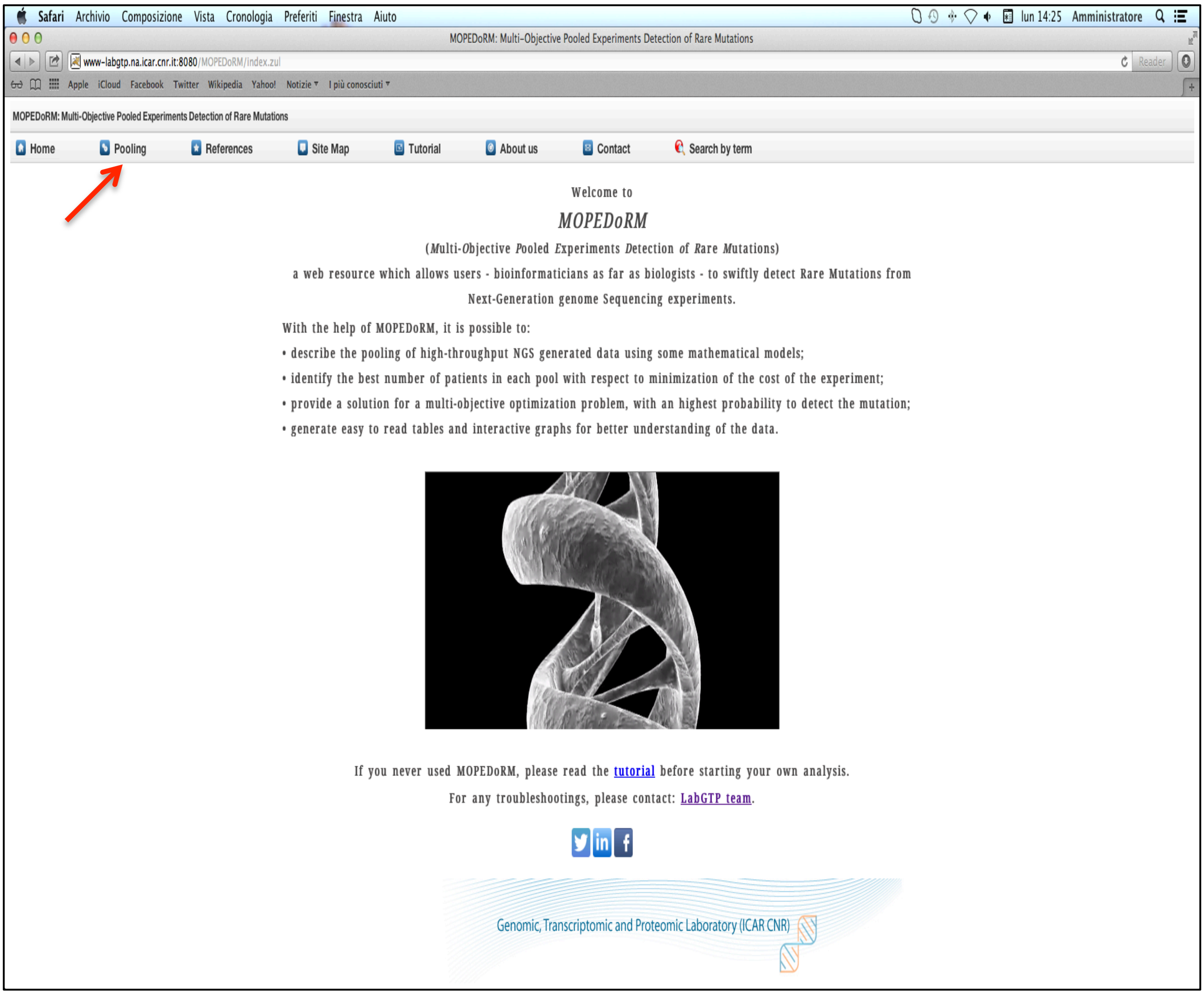

The next page shows a digram portraying the four pooling implemented methods. For sake of simplicity, for the “*Pooling*” section ofonly examples about “*DiagWalks*” algorithm is shown in the next pages. The “*Without Replica* and *With Replica*” subsection contains the same kind of information, and has been developed with the same rationale.

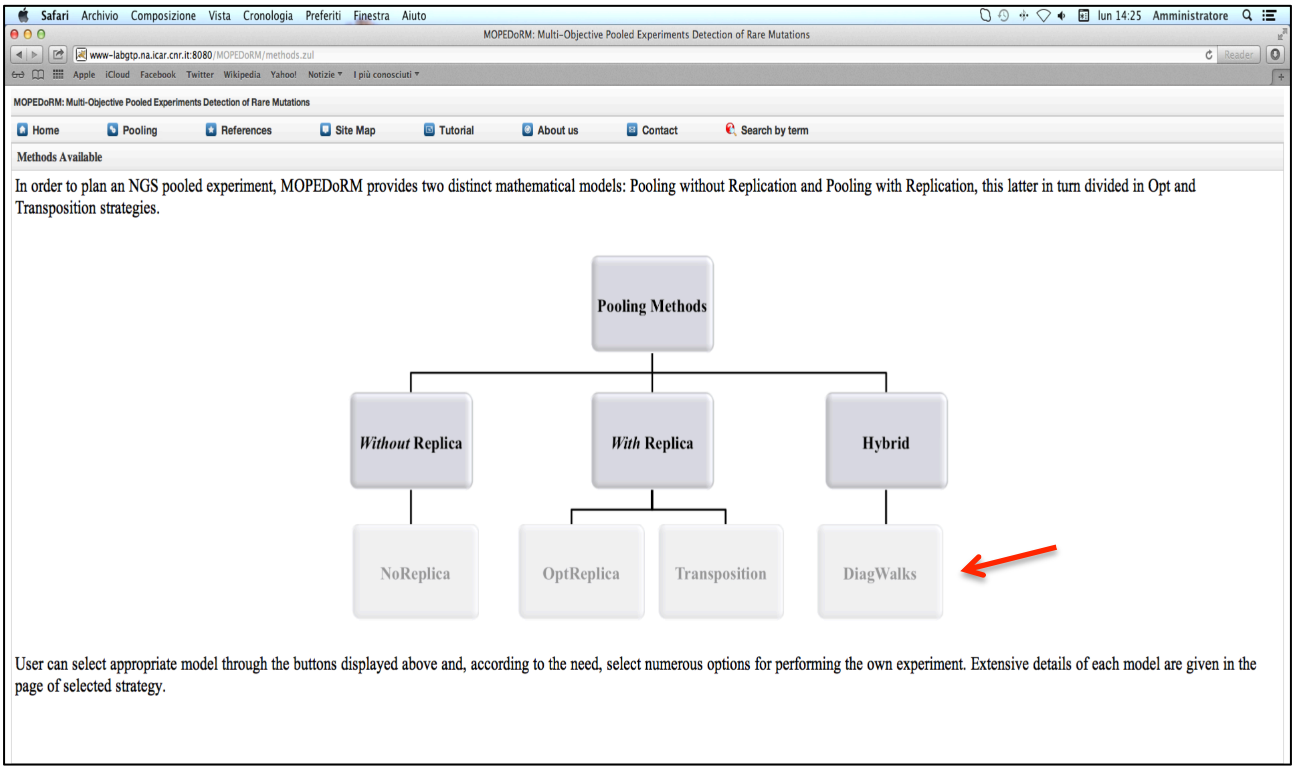

For this case study, the following input data are used:

PARAMETERS	EXPLANATIONS	VALUES
Number of patients	Number of patients	128
Max Pool Size	The maximum number of patients allowed in each pool	1<br>NOTE: When 1 is selected, the program simulates the experiment varying the Max Pool Size for each iteration starting from 1 all the way up to 6.
Number of mutations per patient	How many DNA mutations are expected to be detected	5
Cost SANGER Sequencing Experiments	The price in Euro of each SANGER Sequencing Experiment	8
Cost NGS Sequencing Experiments	The price in Euro of each NGS Sequencing experiment	1000

To start the simulation clicks on the "*Start Run*" button:

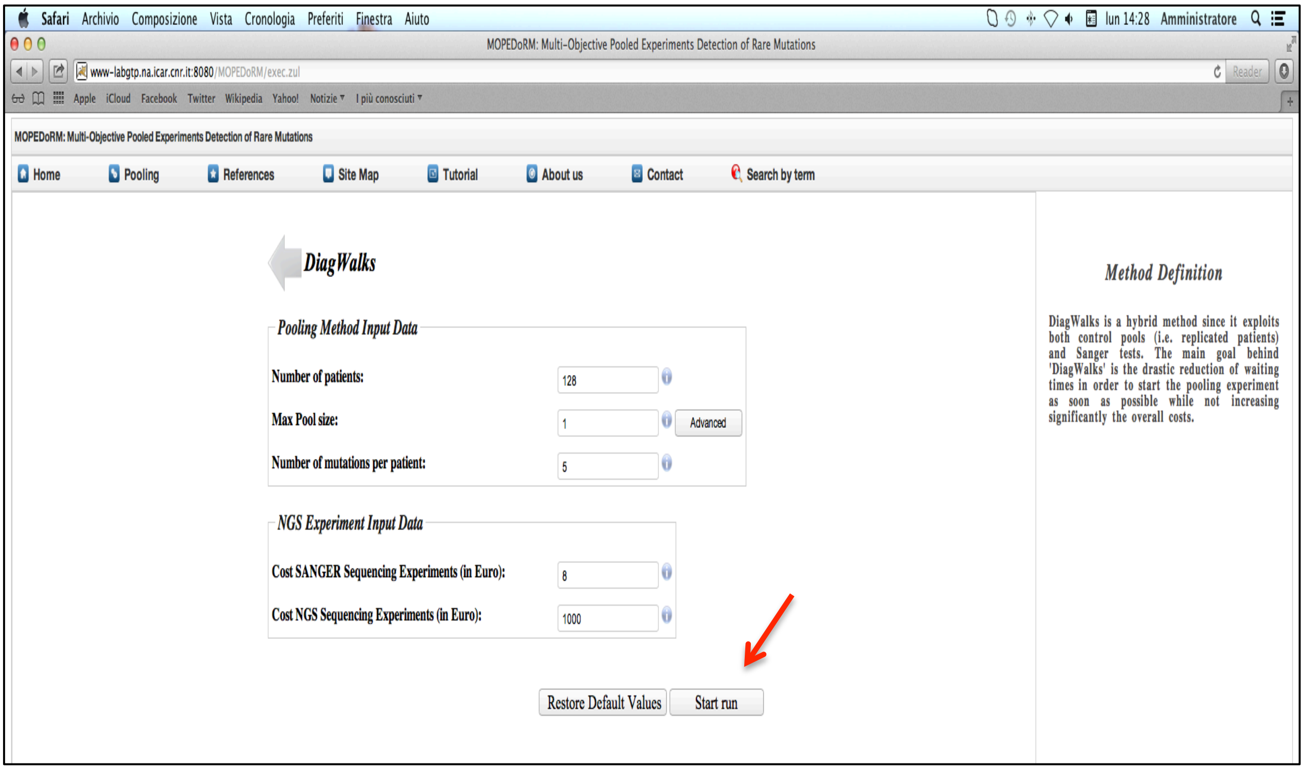

You should wait only few seconds before the task will be successfully completed and the screens that show the computed results will appear.

The resulting page is divided in three subdivision. On the left top corner there is the *Summary of run*, which briefly indicates the input parameters selected before. Under the *Summary of run*, there is a grid indicating the results of each simulation of the NGS experiment using the input data provided before. Whereas, on the right side of the window there is a charts section that indicates the Patient's distribution into pool.

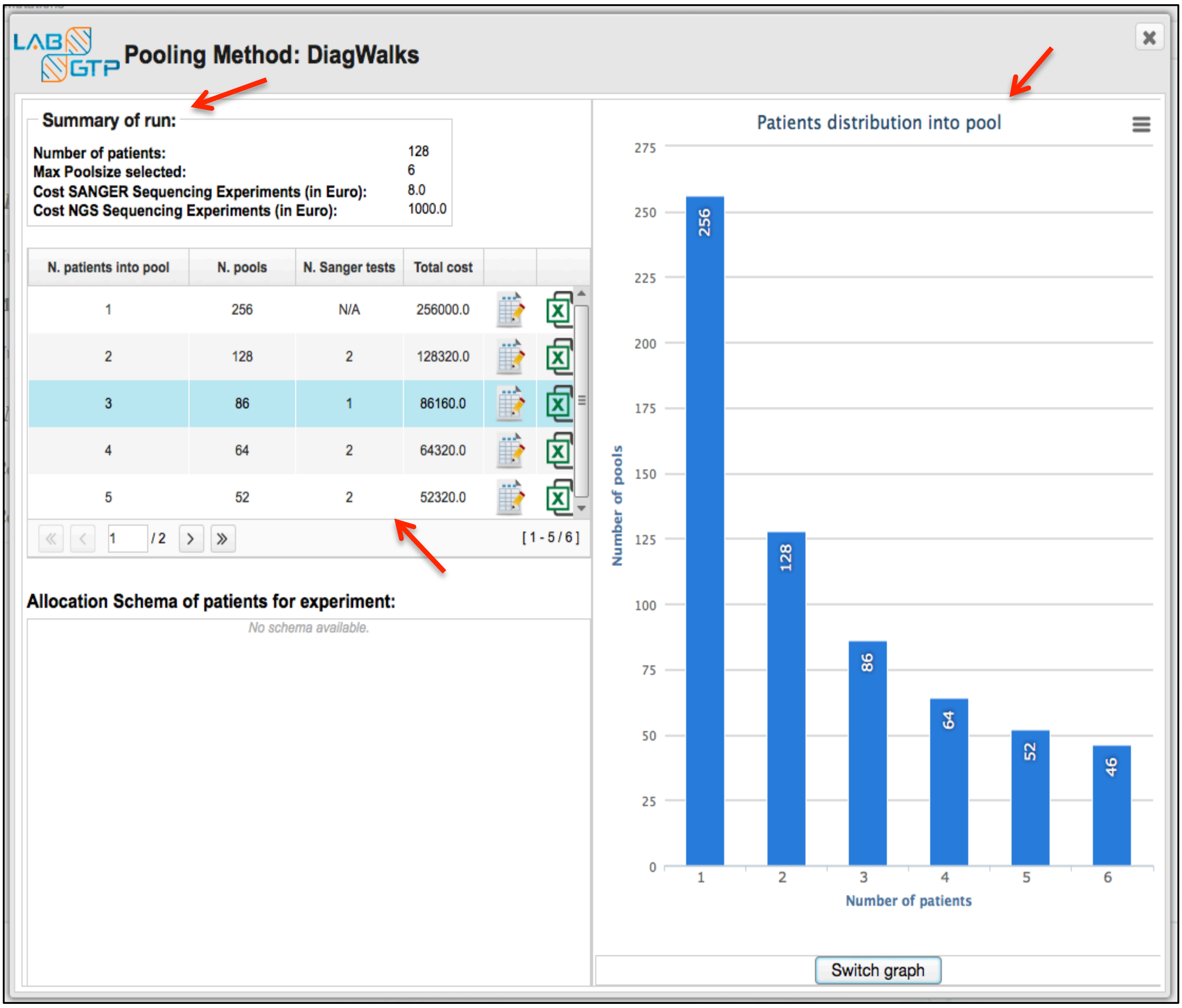

Let's analyze the final report in closer detail. In this specific case, 6 rows are displayed.

PARAMETERS	EXPLANATIONS
N. patients into pool	The number of patients allowed in each pool (i.e. the Max Poolsize computed for that specific simulation)
N. pools	The overall number of pools needed.
N. Sanger tests	The required number of Sanger tests
Total cost	The overall cost (in euros) of the experiment. The lowest computed cost is highlighted in green (in this case, 51280 euros).
Build Allocation Schema	It allows the program to compute and display the arrangement of the patients inside the pools according to the specific input data used for the simulation.
Download Excel:	It allows the user to download an Excel file containing the respective Allocation Schema.

Moreover, the grid is divided in six columns

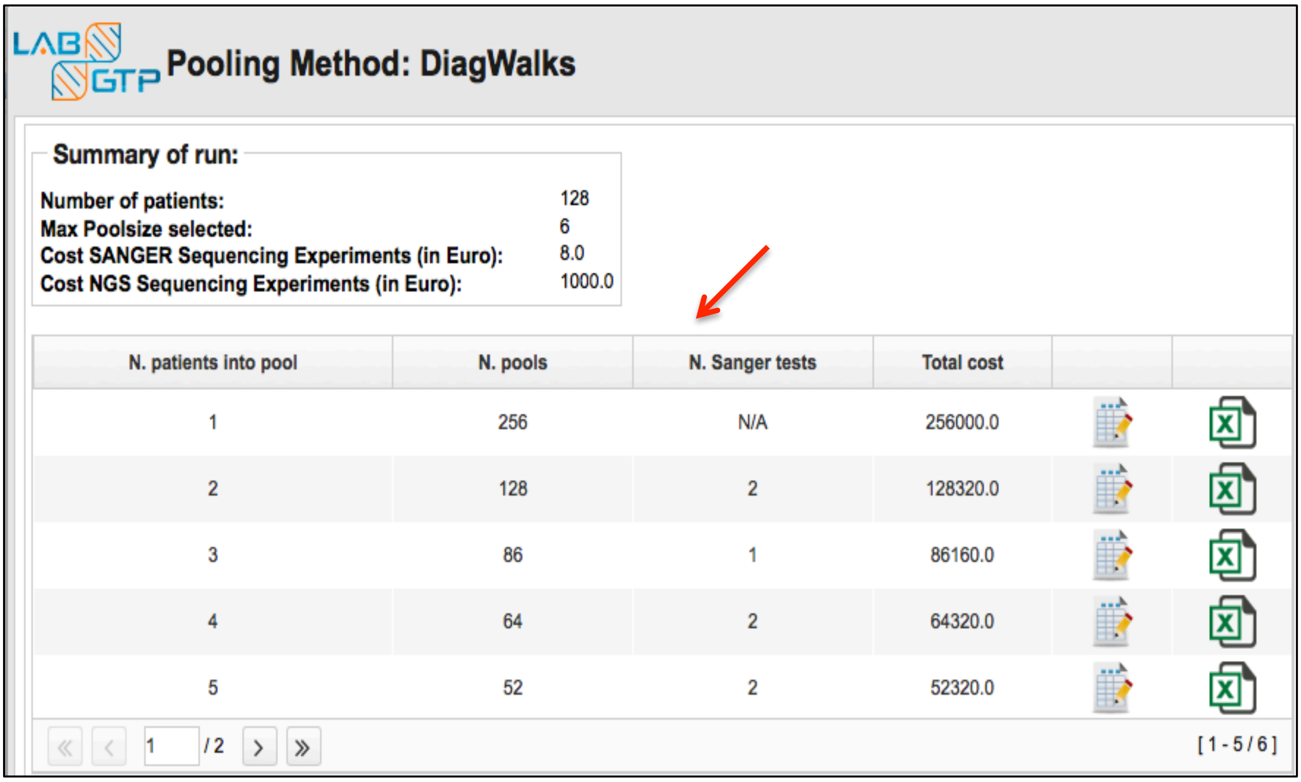

and the best result is reported in green color

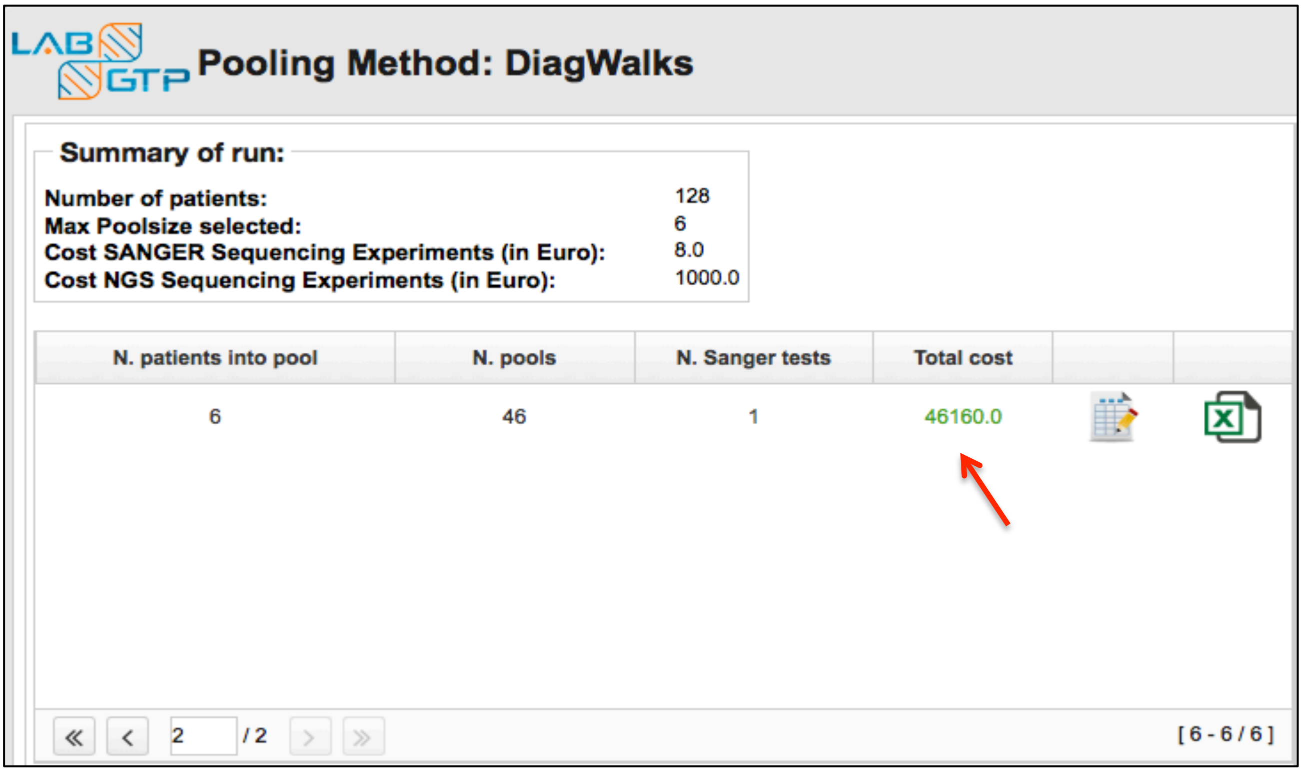

Under the grid outlined before, there is the "*Allocation Schema of patients for experiment*". Once the user clicks on the built Allocation Schema described before, the program will display the Allocation Schema of the patients in this area of the window.

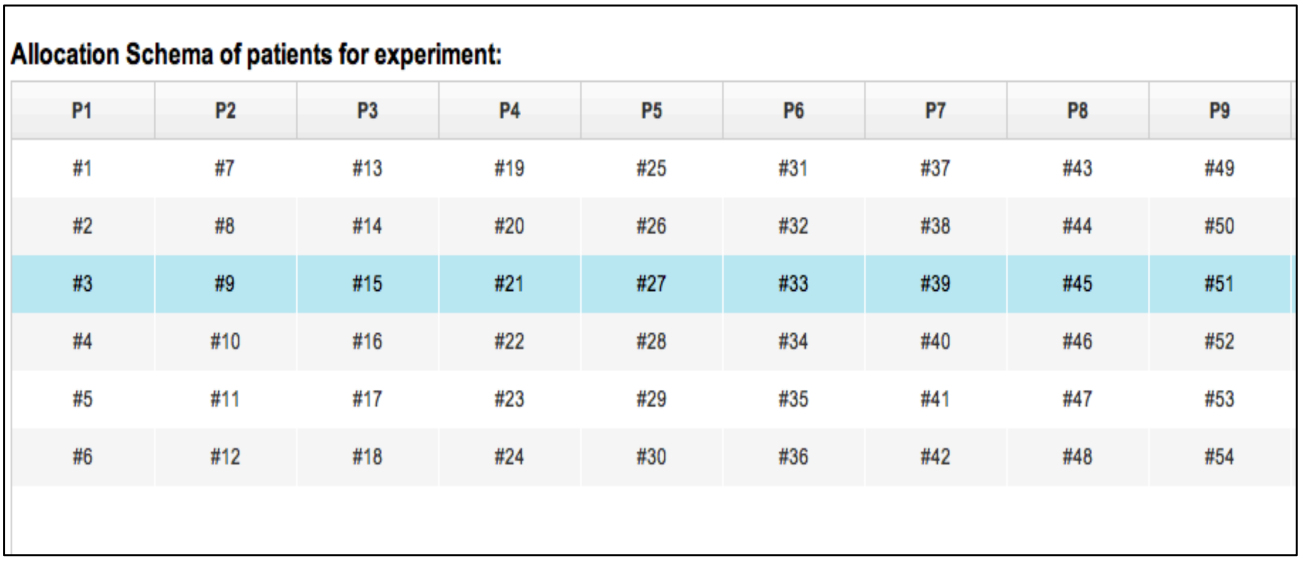

Allocation Schema of patients for experiment:

P1	P2	P3	P4	P5	P6	P7	P8	P9
#1	#7	#13	#19	#25	#31	#37	#43	#49
#2	#8	#14	#20	#26	#32	#38	#44	#50
#3	#9	#15	#21	#27	#33	#39	#45	#51
#4	#10	#16	#22	#28	#34	#40	#46	#52
#5	#11	#17	#23	#29	#35	#41	#47	#53
#6	#12	#18	#24	#30	#36	#42	#48	#54

On the right side of the window, the program automatically draws a column graph called "Patients distribution into pool": on the horizontal axis it indicates the number of patients, on the vertical one the number of pools. This graph shows how the number of patients inside each pool and the required number of pools change for each of the simulations of the experiment.

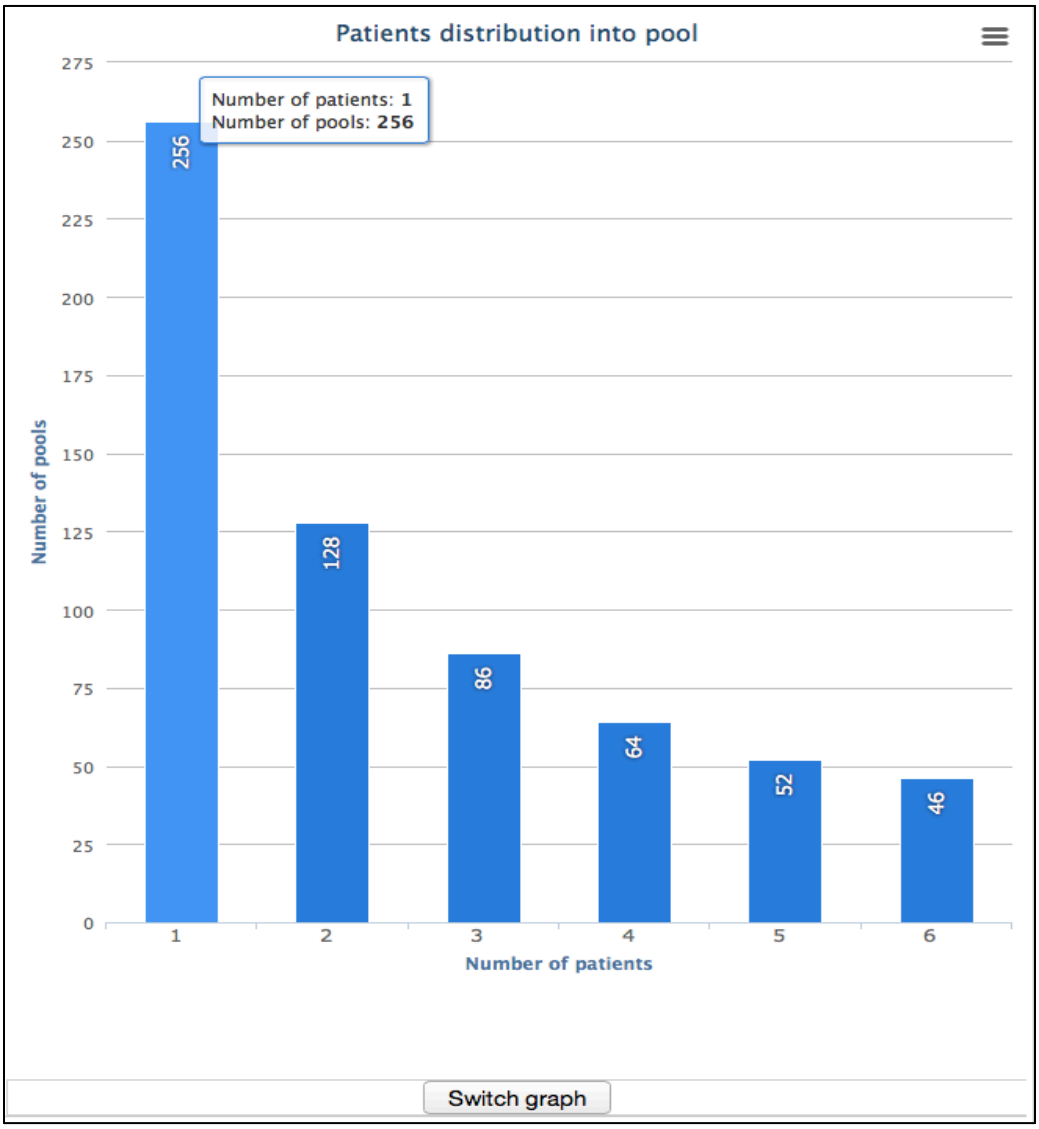

When clicking on the "Switch graph" button, the program draws another graph called "*Trend of NGS cost distribution*": on the horizontal axis it indicates the number of patients, on the vertical one the cost of the experiment. This graph shows how the cost of the experiment changes, for each of the computed iterations, according to the variation of the number of patients, starting from 1 all the way up to the selected input number (in this case, 128).

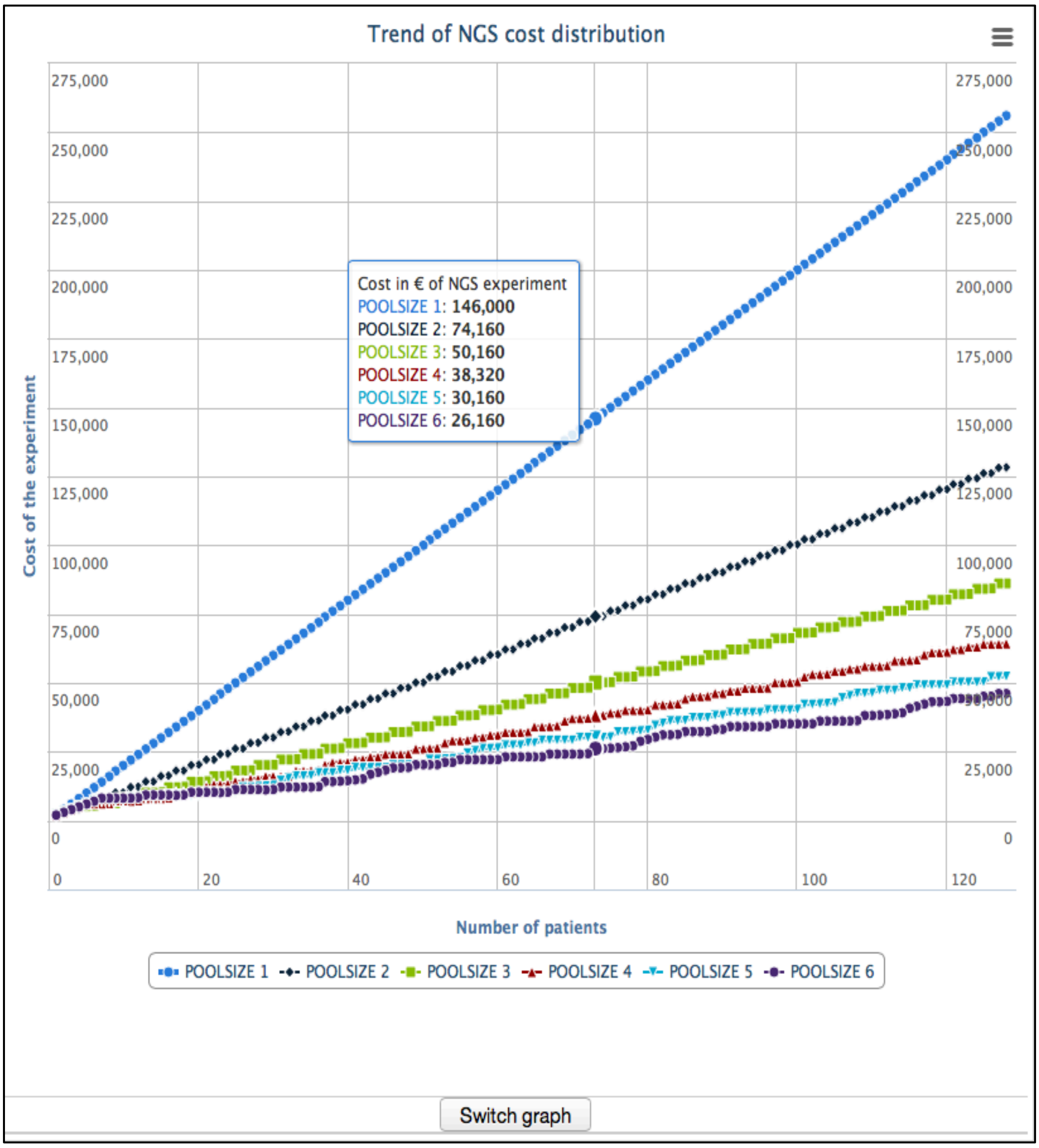

Supplement: Supplementary file 1 — 10.1186/s13104-016-1889-6OPENDoRM user manual. OPENDoRM user’s manual provides detailed case studies with simulated data and illustrates how to use the OPENDoRM algorithms. [file 13104_2016_1889_MOESM1_ESM.pdf]
